# Individual immune cell and cytokine profiles determine platelet-rich plasma composition

**DOI:** 10.1186/s13075-022-02969-6

**Published:** 2023-01-10

**Authors:** Marcel Niemann, Melanie Ort, Luis Lauterbach, Mathias Streitz, Andreas Wilhelm, Gerald Grütz, Florian N. Fleckenstein, Frank Graef, Antje Blankenstein, Simon Reinke, Ulrich Stöckle, Carsten Perka, Georg N. Duda, Sven Geißler, Tobias Winkler, Tazio Maleitzke

**Affiliations:** 1 Charité – Universitätsmedizin Berlin, corporate member of Freie Universität Berlin and Humboldt-Universität zu Berlin, Center for Musculoskeletal Surgery, Augustenburger Platz 1, 13353 Berlin, Germany; 2 Berlin Institute of Health at Charité – Universitätsmedizin Berlin, Julius Wolff Institute, Augustenburger Platz 1, 13353 Berlin, Germany; 3 Department of Biology, Chemistry and Pharmacy, Institute of Chemistry and Biochemistry, Freie Universität Berlin, 14195 Berlin, Germany; 4 Department of Experimental Animal Facilities and Biorisk Management, Friedrich-Loeffler-Institut, Südufer 10, 17493 Greifswald, Insel Riems Germany; 5 Charité – Universitätsmedizin Berlin, corporate member of Freie Universität Berlin and Humboldt-Universität zu Berlin, Department of Diagnostic and Interventional Radiology, Augustenburger Platz 1, 13353 Berlin, Germany; 6 Berlin Institute of Health Center for Regenerative Therapies, Berlin Institute of Health at Charité – Universitätsmedizin Berlin, Augustenburger Platz 1, 13353 Berlin, Germany; 7 Berlin Institute of Health at Charité – Universitätsmedizin Berlin, BIH Biomedical Innovation Academy, BIH Charité Clinician Scientist Program, Anna-Louisa-Karsch-Straße 2, 10178 Berlin, Germany

**Keywords:** Osteoarthritis, Inflammation, Orthobiologics, Regenerative therapies, Immune system

## Abstract

**Objective:**

Platelet-rich plasma (PRP) therapy is increasingly popular to treat musculoskeletal diseases, including tendinopathies and osteoarthritis (OA). To date, it remains unclear to which extent PRP compositions are determined by the immune cell and cytokine profile of individuals or by the preparation method. To investigate this, we compared leukocyte and cytokine distributions of different PRP products to donor blood samples and assessed the effect of pro-inflammatory cytokines on chondrocytes.

**Design:**

For each of three PRP preparations (ACP®, Angel™, and nSTRIDE® APS), products were derived using whole blood samples from twelve healthy donors. The cellular composition of PRP products was analyzed by flow cytometry using DURAClone antibody panels (DURAClone IM Phenotyping Basic and DURAClone IM T Cell Subsets). The MESO QuickPlex SQ 120 system was used to assess cytokine profiles (V-PLEX Proinflammatory Panel 1 Human Kit, Meso Scale Discovery). Primary human chondrocyte 2D and 3D in vitro cultures were exposed to recombinant IFN-γ and TNF-α. Proliferation and chondrogenic differentiation were quantitatively assessed.

**Results:**

All three PRP products showed elevated portions of leukocytes compared to baseline levels in donor blood. Furthermore, the pro-inflammatory cytokines IFN-γ and TNF-α were significantly increased in nSTRIDE® APS samples compared to donor blood and other PRP products. The characteristics of all other cytokines and immune cells from the donor blood, including pro-inflammatory T cell subsets, were maintained in all PRP products. Chondrocyte proliferation was impaired by IFN-γ and enhanced by TNF-α treatment. Differentiation and cartilage formation were compromised upon treatment with both cytokines, resulting in altered messenger ribonucleic acid (mRNA) expression of collagen type 1A1 (*COL1A1*), *COL2A1*, and aggrecan (*ACAN*) as well as reduced proteoglycan content.

**Conclusions:**

Individuals with elevated levels of cells with pro-inflammatory properties maintain these in the final PRP products. The concentration of pro-inflammatory cytokines strongly varies between PRP products. These observations may help to unravel the previously described heterogeneous response to PRP in OA therapy, especially as IFN-γ and TNF-α impacted primary chondrocyte proliferation and their characteristic gene expression profile. Both the individual’s immune profile and the concentration method appear to impact the final PRP product.

**Trial registration:**

This study was prospectively registered in the Deutsches Register Klinischer Studien (DRKS) on 4 November 2021 (registration number DRKS00026175).

**Supplementary Information:**

The online version contains supplementary material available at 10.1186/s13075-022-02969-6.

## Introduction

The Food and Drug Administration (FDA) defines platelet-rich plasma (PRP) as a centrifuged plasma product obtained by an uninterrupted venipuncture with at least 250,000 platelets per μl [1]. Despite limited evidence for its efficacy, PRP is widely used to treat acute musculoskeletal injuries including rotator cuff tears and chronic degenerative joint disorders like osteoarthritis (OA) [2]. A wide variety of PRP products is currently available. Products differ in terms of the manufacturing process, their cellular (e.g., platelets and leukocytes) and molecular (e.g., growth factors and anti-inflammatory cytokines) compositions, and their exogenous activation [3]. It is assumed that PRP products have anti-inflammatory and distinct immunomodulatory properties [4], which can be adjusted to the individual needs of the patient and the treated disease.

It is currently unclear which patients respond well to PRP treatment, and to which extent their response to treatment is related to the cellular and molecular composition of the initial blood sample from which the PRP product is derived. Previous studies have shown that the concentration of pro-angiogenic growth factors and catabolic proteases is positively correlated with leukocyte counts in the PRP product [5]. Among leukocytes, granulocytes and lymphocytes are known to be important modulators of endogenous regeneration [6], but their concentration in peripheral blood is strongly dependent on donor age [7], sex [8], and physical activity [9]. Locally elevated granulocyte counts appear to be crucial for facilitating fracture and wound healing [10–12] and elevated granulocyte concentrations in the peripheral blood are being discussed as predictive biomarkers for OA severity [13]. Furthermore, elevated granulocyte counts have previously been correlated with an increased incidence of osteonecrosis of the femoral head [14].

Each individual has a unique adaptive immune profile that reflects the personal immune experience resulting from exposure to different antigens throughout life. Whether individual lymphocyte signatures or corresponding circulating cytokine levels are directly transferred into PRP products is unknown. PRP compositions differ between women and men [15] and are dependent on the PRP manufacturing system [4]. Thus, it is of great importance to clarify whether the cellular and cytokine profiles of individuals are transferred into the PRP product. Furthermore, uncovering the impact of the manufacturing process on PRP compositions is necessary, when considering individualized treatment strategies.

We hypothesize that the individual profile of distinct leukocyte subsets in the donor blood determines the PRP composition. We assume that the degree of leukocyte and cytokine enrichment depends on the specific PRP manufacturing process. To address this, we systematically analyzed the cellular and molecular composition of PRP products from three commercially available systems (ACP®, Angel™, and nSTRIDE® APS) and compared them to the corresponding blood samples from healthy donors. Furthermore, we explored the role of selected pro-inflammatory cytokines on cartilage, by exposing primary human chondrocytes to IFN-γ and TNF-α.

## Materials and methods

This study was conducted following local ethics committee approval (Ethikkommission Charité – Universitätsmedizin Berlin, approval numbers EA2/218/21 and EA1/146/21) and in accordance with the Declaration of Helsinki. All participants have given written informed consent to study participation. Sample size and effect sizes were estimated using nQuery (nQuery + nTerim, version 4.0, GraphPad Software DBA Statistical Solutions, San Diego, CA, United States of America (USA)). Estimations were based on a confidence level of 95% and a power of 80% to detect effects.

### Platelet-rich plasma production

Twelve healthy participants (five female) were recruited for study inclusion and blood samples of 115 ml were taken from each participant to produce the PRP products.

A maximum volume of 15 ml of donor blood was used for the ACP® system (Autologous Conditioned Plasma, ACP®, Arthrex GmbH, Naples, FL, USA). Without adding an anticoagulant, the blood was directly drawn into the double syringe provided by the manufacturer. Using a Hettich Centrifuge (Rotofix 32 A 220 V, Hettich GmbH & Co. oHG, Kirchlengern, NRW, Germany), each sample was centrifuged at 1500 revolutions per minute (rpm) for 5 min. Double syringes were handled according to the manufacturer’s instructions [16]. The resulting PRP product was left in the inner syringe for immediate processing and analysis.

For the Angel™ system (Arthrex Angel™, Arthrex GmbH, Naples, FL, USA), 40 ml of blood was drawn into the syringe provided in the manufacturer’s kit. An acid-citrate-dextrose solution (ACD-A 30 ml, Zimmer Biomet Holdings, Warsaw, IN, USA) was added to the syringe in a 1:7 ratio to the blood volume. For further PRP preparation, we followed the manufacturer’s recommendations [17]. The Angel™ centrifuge was set to reach a hematocrit of 2% in the final product. PRP was collected in a syringe for immediate processing and analysis.

The nSTRIDE® APS (nSTRIDE® Activated Protein Solution, Zimmer Biomet Holdings, Warsaw, IN, USA) was manufactured by mixing 55 ml of donor blood and 5 ml of ACD-A (ACD-A 30 ml, Zimmer Biomet Holdings) in the provided syringe, which was then placed in the cell separator containers and centrifuged at 3200 rpm for 15 min. The supernatant was prepared according to the manufacturer’s protocol [18] and the resulting fraction was again centrifuged at 2000 rpm for 2 min. The final product was transferred into a syringe which was then used for immediate processing and analysis.

The remaining 5 ml of the donor blood was used as the control for all subsequent laboratory analyses. Samples were collected in standard ethylenediaminetetraacetic acid (EDTA) tubes (Vacuette® EDTA tubes, Greiner Bio-One, Greiner Group AG, Kremsmünbster, Austria) for immediate processing and analysis.

### Flow cytometry analysis of immune cell composition

DURAClone antibody panels were used for flow cytometry. For basic immune subset identification, CD16-FITC, CD56-PE, CD19-ECD, CD14-PC7, CD4-APC, CD8-A700, CD3-APC-A750, and CD45-KrO were assessed (DURAClone IM Phenotyping Basic, kit #B53309, Beckman Coulter, Washington, D. C., USA). For identification of T cell subsets, CD45RA-FITC, CD197-PE, CD28-ECD, CD279-PC5.5, CD27-PC7, CD4-APC, CD8-A700, CD3-APC-A750, CD57-PB, and CD45-KrO were measured (DURAClone IM T Cell Subsets, kit #B53328, Beckman Coulter).

For each sample, 100 μl of donor blood or respective PRP was added into the DURAClone tubes. Following staining in the dark at room temperature, 2 ml of VersaLyse erylysis buffer was added and tubes were centrifuged in an Eppendorf centrifuge (Centrifuge 5810 R, Eppendorf SE, Hamburg, Germany) at 200 g for 5 min. While the supernatant was separately stored for cytokine profiling, 3 ml phosphate-buffered saline (PBS) was added to the tubes. The samples were centrifuged at 200 g for 5 min and respective supernatants were discarded. Each cell pellet was resuspended in 200 μl buffer (containing 1× PBS with 5% v/v of fetal bovine serum, 2 mM EDTA, and 2 mM sodium azide).

Samples were analyzed using a Navios EX cytometer (Navios EX, Beckman Coulter). Acquired immune cells were described as follows: leukocytes (CD45^+^), granulocytes, identified using forward and sideward scatters, and depicted as NGr^+^ (CD45^+^FSC/SSC), while lymphocytes and monocytes were depicted as NGr^−^ (CD45^+^FSC/SSC), monocytes (CD45^+^CD14^+^), classical monocytes (CD45^+^CD14^high^CD16^−^), non-classical monocytes (CD45^+^CD14^dim^CD16^+^), intermediate monocytes (CD45^+^CD14^high^CD16^+^), B cells (CD45^+^CD14^−^CD3^−^CD19^+^), natural killer (NK) cells (CD45^+^CD14^−^CD19^−^CD3^−^CD56^+^), T cells (CD45^+^CD14^−^CD19^−^CD3^+^), T helper cells (CD45^+^CD14^−^CD19^−^CD3^+^CD8^−^CD4^+^), and cytotoxic T cells (CD45^+^CD14^−^CD19^−^CD3^+^CD4^−^CD8^+^). CD4^+^ and CD8^+^ T cells were further separated into central memory (CD45RA^−^CD197^+^), naive (CD45RA^+^CD197^+^), effector memory (CD45RA^−^CD197^−^), and terminally differentiated effector memory (TEMRA) T cells (CD45RA^+^CD197^−^). See Supplementary Fig. S1 for the detailed gating strategy.

### Determination of cytokine levels with Meso Scale multiplexing immunoassays

Supernatants of PRP products and donor blood samples were stored at −80 °C until needed for cytokine measurements. The concept of Meso Scale multiplexing immunoassays is based on the sandwich enzyme-linked immunosorbent assay (ELISA) principle. Commercially available capture antibodies are precoated on conductive plates to which the samples are applied. The data output was analyzed using the MESO QuickPlex SQ 120 system proinflammatory cytokine panel (V-PLEX Proinflammatory Panel 1 Human Kit, Meso Scale Discovery (MSD), Meso Scale Technologies, LLC, Rockville, MD, USA) which quantifies the concentrations of interferon gamma (IFN-γ), interleukin 1β (IL-1β), IL-2, IL-4, IL-6, IL-8, IL-10, IL-12p70, IL-13, and tumor necrosis factor alpha (TNF-α) [19].

### Cell culture experiments

#### Isolation of chondrocytes

Cartilage was obtained from patients undergoing total knee replacement surgery for knee OA (*n*=3). Cartilage samples were cut into small pieces, added to the pre-warmed basal medium (low-glucose Dulbecco’s modified Eagle medium [LG-DMEM, Thermo Fisher Scientific Inc., Waltham, MA, USA], 1% penicillin-streptomycin [PenStrep, 10.000 U/ml, Thermo Fisher Scientific Inc.], and 1% GlutaMAX [GlutaMAX™, Thermo Fisher Scientific Inc.]) supplemented with 1mg/ml collagenase type II (Thermo Fisher Scientific Inc.), and incubated at 37 °C for 12–24 h at 5% CO_2_. Samples were spun down at 300 g for 5 min, supernatants were discarded, and fresh expansion medium (basal medium 10% fetal bovine serum [FBS, Thermo Fisher Scientific Inc.]) was added. The medium was changed every 4 days and cells were grown for 2 to 3 weeks. For all in vitro experiments, cells were used in passages two to three.

#### 2D cell culture and TNF-α and IFN-γ treatment

Primary human chondrocytes of three different patients were seeded at 30% confluency with 3×10^3^ cells per well in 48-well plates. After 24 h in the expansion medium, cells were treated with minimal medium containing LG-DMEM, 2% human male AB serum (Merck KGaA, Darmstadt, Germany), 1% PenStrep, 1% GlutaMAX, and different concentrations of TNF-α (recombinant human TNF-α, reference 55418, BD Biosciences, Franklin Lakes, NJ, USA) or IFN-γ (animal-free recombinant human IFN-γ, reference AF-300-02, PeproTech, Thermo Fisher Scientific Inc) (0, 1, 10, and 100 ng/ml, respectively). Proliferation, metabolic activity, and lactate dehydrogenase (LDH cytotoxicity detection kit, reference 4744934001, Sigma-Aldrich, St. Louis, MO, USA) release were determined after 7 days upon stimulation as described below.

#### 3D chondrocyte disc chondrogenesis and TNF-α and IFN-γ treatment

3D chondrogenic differentiation was performed as previously described with minor modifications [20]. Briefly, 24-well-plate tissue culture inserts (Millicell®, Merck KGaA) were coated with 80 μl of rat collagen type I (0.2 μg/ml, Thermo Fisher Scientific Inc.) per insert and incubated for 2 h. The remaining supernatant was removed and inserts were washed with PBS. 0.5×10^6^ cells per insert were washed with PBS and centrifuged at 300 g for 5 min and resuspended in 100 μl chondrogenic differentiation medium (CDM: 0.1 μM dexamethasone, 50 μg/ml l-ascorbic acid 2-phosphate, 40 μg/ml l-proline, 10 mg/ml Natriumpyruvat, 0.5 mg/ml insulin-transferrin-sodium selenite media supplement (ITS), 125 mg/ml bovine serum albumin (BSA), 5.35 mg/ml linoleic acid, 10 ng/ml transforming growth factor β1 (TGF-β1), and 1% PenStrep in LG-DMEM). Cells were added to inserts, centrifuged at 300 g for 5 min, and the lower compartment in the well filled with 500 μl CDM. Three days after seeding, 10 ng/ml of TNF-α or IFN-γ was added to the CDM of the indicated cultures to analyze early effects of pro-inflammatory cytokines on chondrogenic differentiation (early inflammation approach). To investigate the impact of a pro-inflammatory environment on chondrocyte phenotype stability, 10 ng/ml of TNF-α was added to the CDM of the indicated cultures on day 14 after differentiation induction (late TNF-α). All cell cultures were incubated at 37 °C and 5% CO_2_ for 28 days and the medium was changed three times per week. The formed discs were then removed from the transwell and used for further real-time quantitative polymerase chain reaction (qPCR) and proteoglycan analyses.

### Cell proliferation and metabolic activity assays

Cell proliferation was determined using the fluorescent-based CyQuant™ Cell Proliferation assay (Thermo Fisher Scientific Inc.) according to the manufacturer’s recommendation and as previously described [21]. Briefly, following a freeze-thaw cycle, cells were incubated with a detection buffer for 5 min at room temperature and analyzed with a plate reader (Infinite M PLEX MONO 200, Tecan, Männedorf, Switzerland) at 480/520 nm. Chondrocyte growth kinetics were quantified by calculating population doublings (PD) at day 7 based on the following equation: PD = log_2_(*N*_day7_/*N*_day_0). Metabolic activity was assessed with PrestoBlue™ (Thermo Fisher Scientific Inc.), which is a resazurin-based cell assay. Chondrocytes were incubated with 1:10 PrestoBlue™ solution for 1 h at 37 °C. Fluorescence was measured at 560/590 nm (excitation/emission) using a Tecan plate reader. PrestoBlue™ data was normalized to CyQuant™ data sets. LDH activity was used to determine cell death. Activity was measured in the supernatant of all wells in 3D and 2D cultures to monitor cytotoxicity over time. Absorbance values directly correlate with the amount of formed formazan salt which is proportional to the number of lysed cells.

#### Cell and disc digestion for RNA isolation and qPCR

Chondrocyte discs were mixed with 250 μl Trizol (TRI Reagent, reference R2050-1-200, Zymo Research, Irvine, CA, USA) in special tubes with Minilys beads (VWR Life Science, Bertin Corp., Rockville, MD, USA), homogenized and pooled. A total of 250 μl of chloroform (Merck KGaA, Darmstadt, Germany) was added and samples were centrifuged at 12,000 g for 10 min at 4 °C. The aqueous phase (~450 μl) was added to a fresh tube containing 10 μg of glycogen (Merck KGaA). Samples were then mixed with an equal amount of isopropanol (Merck KGaA), incubated for 10 min, and centrifuged at 12,000 g for 10 min at 4 °C. Supernatants were removed, and RNA pellets washed with 75% ice-cold ethanol (Merck KGaA) and briefly vortexed. A last centrifugation step at 7500 g for 2 min at 4 °C was performed followed by air drying of the pellets and resuspension in 20 μl H_2_O.

#### Primers and qPCR

For qPCR, approximately 500 ng ribonucleic acid (RNA) were reverse transcribed (iScript™ cDNA Synthesis Kit, Bio-Rad Laboratories Inc., Hercules, CA, USA). For all discs, qPCR was performed using primers for aggrecan (*ACAN*), collagen type 1 (*COL1A1*), collagen type 2 (*COL2A1*), matrix metalloproteinase 3 (*MMP3*), *MMP9*, and *MMP13* as previously described [21]. Briefly, the thermal profile used was 95 °C for 10 min and 95, 60, and 72 °C for 40 s, respectively, 95–65°C per cycle. The primer list can be found in Supplementary Table S1.

#### Proteoglycan assay and protein quantification with Bradford assay

For proteoglycan analysis, discs were digested in tubes with Minilys beads (VWR Life Science) as described above, but with 150 μl proteoglycan extraction buffer (Merck KgaA). Then, 25 μl of the digested samples were mixed with 250 μl dimethylmethylene blue (DMMB, Merck KGaA) assay reagent and quantified with a Tecan plate reader at 516 nm. For Bradford assays, 5 μl of each sample mixed with 250 μl dye (Bio-Rad Laboratories Inc.), vortexed, and stored for 5 min at room temperature in dark and read with a Tecan plate reader at 595 nm.

### Analysis and statistics

Flow cytometry data files were analyzed using Kaluza Analysis (Kaluza Analysis, version 2.1, Beckman Coulter, Washington, D. C., USA). MSD data, including controls and standards, were analyzed using MSD Workbench (MSD Workbench, version 4.0, Meso Scale Technologies, LLC, Rockville, MD, USA).

Statistical analysis was performed using GraphPad Prism (GraphPad Prism for macOS, version 9.3.0, GraphPad Holdings, LLC, San Diego, CA, USA). Donor blood and PRP product samples were defined as dependent samples. Accordingly, the Friedman test was used and a post hoc analysis was performed with Dunn’s correction for multiple testing. We used the Pearson correlation to assess correlations between samples. For 2D and 3D cell culture analysis, a one-way ANOVA including Dunn’s correction for multiple testing was used. Unless stated otherwise, frequencies are represented as numbers (portion of the whole [%]) and not normally distributed values are represented as median (IQR [25th percentile, 75th percentile]). All *p*-values are two-tailed and *p*-values ≤ 0.05 were considered statistically significant.

## Results

The median age of this population was 31 years (range 26–51). Five participants (41.7%) were females, none of the participants showed signs of systemic or local infections at the time of PRP preparation, and none had any chronic medical condition.

### Individual leukocyte profile is maintained in PRP products

**Fig. 1:**
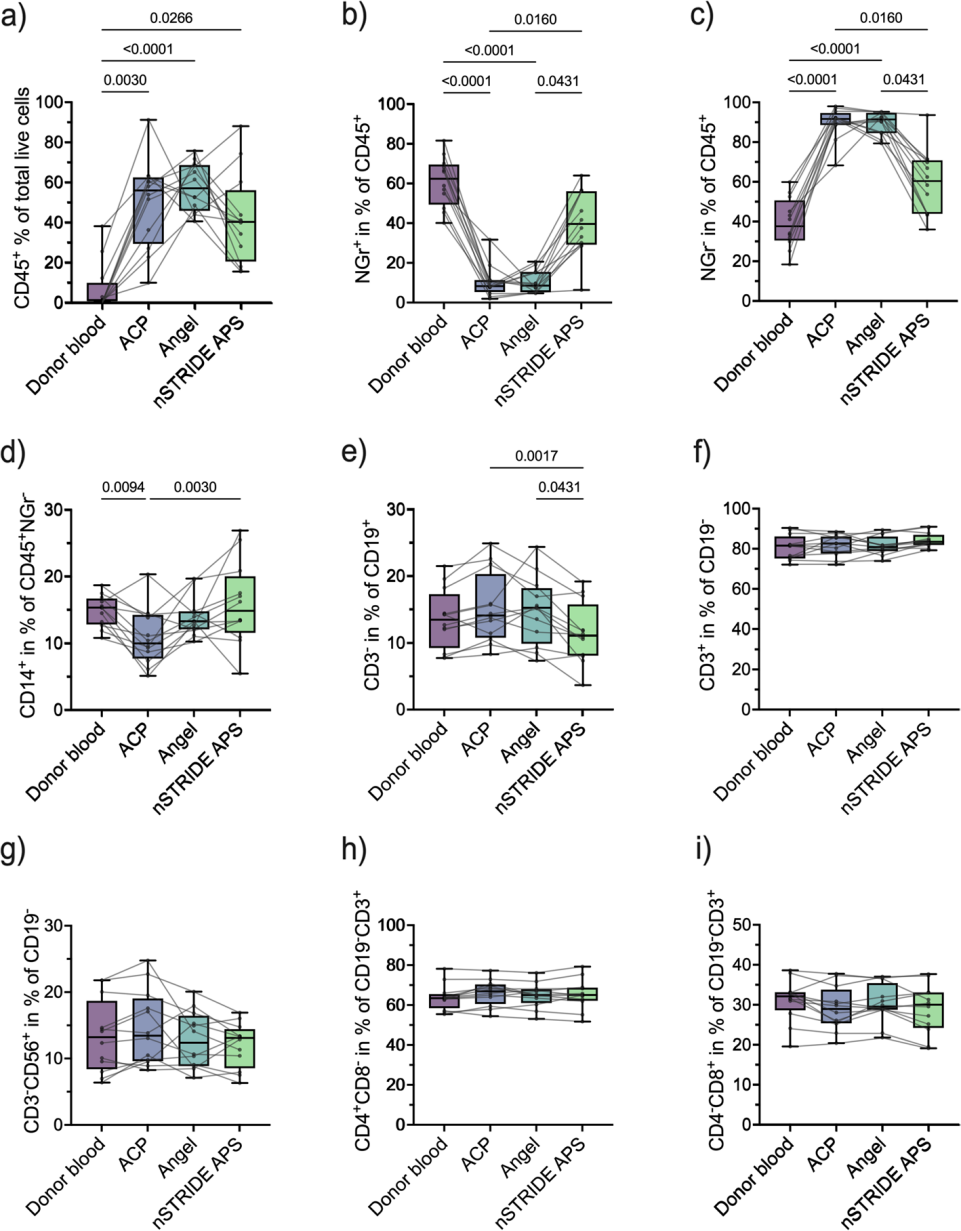
Cellular composition of different PRP products and corresponding donor blood samples. **a** Proportion of leukocytes (CD45^+^), **b** granulocytes (CD45^+^FSC/SSC NGr^+^), **c** lymphocytes and monocytes (CD45^+^FSC/SSC NGr^−^), **d** monocytes (CD45^+^CD14^+^), **e** B cells (CD45^+^CD14^−^CD19^+^CD3^−^), **f** T cells (CD45^+^CD14^−^LyCD19^−^CD3^+^), **g** NK cells (CD45^+^CD14^−^CD19^-^CD3^−^CD56^+^), **h** CD4^+^ T helper cells (CD45^+^CD14^−^CD19^−^CD3^+^CD4^+^CD8^−^), and **i** CD8^+^ cytotoxic T cells (CD45^+^CD14^−^CD19^−^CD3^+^CD4^−^CD8^+^) in donor blood compared to PRP samples. Abbreviations: NK cells, natural killer cells; PRP, platelet-rich plasma

To determine the cellular composition of the different PRP products and the corresponding donor blood, we first performed a basic characterization of granulocytes, the major lymphocyte subsets, and monocyte subpopulations. This analysis revealed a significant enrichment of leukocytes in all PRP products (ACP®: 55.9% [IQR 29.2, 62.4], *p* = 0.003; Angel™: 57.0% [IQR 45.7, 68.6], *p* < 0.0001; nSTRIDE® APS: 40.2% [IQR 20.4, 56.0], *p* = 0.027) compared to donor blood (1.1% [IQR 0.7, 9.7]) (Fig. 1a). In all but one product, the increased leukocyte levels were accompanied by a decrease in the relative amount of granulocytes compared to donor blood (NGr^+^) (donor blood: 62.3% [IQR 49.3, 69.5]; ACP®: 8.2% [IQR 5.3, 11.3], *p* < 0.0001; Angel™: 8.5% [IQR 5.2, 15.4], *p* < 0.0001; nSTRIDE® APS: 39.6% [IQR 29.1, 56.0], *p* = 0.347) (Fig. 1b), while monocytes and lymphocytes (NGr^−^) were concentrated in all PRP products compared to the corresponding values in the blood (donor blood: 37.7% [IQR 30.5, 50.7]; ACP®: 91.8% [IQR 88.7, 94.7], *p* < 0.0001; Angel™: 91.4% [IQR 84.6, 94.8], *p* < 0.0001; nSTRIDE® APS: 60.4% [IQR 44.0, 70.9], *p* = 0.347) (Fig. 1c).

Although the relative amount of NGr^−^CD14^+^ monocytes was slightly reduced in the different PRP products compared to donor blood (donor blood: 15.3% [IQR 12.8, 16.6]; ACP®: 10.0% [IQR 7.7, 14.2], *p* = 0.009; Angel™: 13.2% [IQR 12.0, 14.7], *p* = 0.683; nSTRIDE® APS: 14.8% [IQR 11.5, 20.0], *p* > 0.999) (Fig. 1d), the changes of the donor-specific ratio between the monocyte subpopulations were more pronounced. The non-classical subpopulation was significantly reduced in nSTRIDE® APS (2.4% [IQR 1.7, 3.3]) compared to donor blood and the other products (donor blood: 6.7% [IQR 4.8, 7.8], *p* = 0.005; ACP®: 5.8% [IQR 4.3, 9.9], *p* = 0.009; Angel™: 7.6% [IQR 4.2, 11.2], *p* = 0.0001) (Supplementary Fig. S2a). The classical subpopulation was significantly reduced in ACP® (71.8% [IQR 51.0, 74.8]) compared to donor blood (81.8% [IQR 77.1, 87.4], *p* = 0.0009) and nSTRIDE® APS (79.8% [IQR 74.2, 86.4], *p* = 0.027), as well as in Angel™ (68.1% [IQR 62.0, 75.2], *p* = 0.003) compared to donor blood (Supplementary Fig. S2b). Lastly, the intermediate subpopulation was significantly concentrated in nSTRIDE® APS (8.9% [IQR 5.9, 11.7]) compared to donor blood and the other PRP products (donor blood: 3.1% [IQR 2.0, 4.6], *p* = 0.043; ACP®: 2.6% [IQR 1.8, 4.8], *p* = 0.043; Angel™: 2.0% [IQR 1.0, 3.0], *p* < 0.0001) (Supplementary Fig. S2c).

The levels of CD19^+^ B cells were significantly lower in nSTRIDE® APS (11.1% [IQR 8.1, 15.7]) than in the other two PRP products (ACP®: 14.0% [IQR 10.8, 20.3], *p* = 0.002; Angel™: 15.2% [IQR 9.9, 18.1], *p* = 0.043), but no statistical difference was observed between the PRP products and donor blood (13.4% [IQR 9.2, 17.2]) (Fig. 1e). The correlation analysis revealed that the relative amount of CD19^+^ B cells within the different PRP products was consistent with baseline donor blood levels (Supplementary Fig. S3a). No significant difference in relative proportions of CD3^+^ T cells (donor blood: 81.7% [IQR 75.3, 86.2]; ACP®: 82.7% [IQR 77.7, 86.2]; Angel™: 81.0% [IQR 78.9, 86.2]; nSTRIDE® APS: 83.6% [IQR 82.0, 87.0]) (Fig. 1f) or CD3^−^CD56^+^ NK cells (donor blood: 13.2% [IQR 8.4, 18.7]; ACP®: 13.5% [IQR 9.6, 19.0]; Angel™: 12.4% [IQR 8.9, 16.4]; nSTRIDE® APS: 13.1% [IQR 8.5, 14.2]) (Fig. 1g) could be found between the PRP products and donor blood. Correlation analysis showed that relative proportions of CD3^+^ T cells and CD3^−^CD56^+^ NK cells were consistent with baseline levels in donor blood for ACP® and Angel™, but not for nSTRIDE® APS (Supplementary Fig. S3b-c).

To investigate the extent to which the adaptive immune profile of the donor is maintained in PRP products, the composition of distinct T cell subsets was analyzed as a central component of individual immunity. No statistical difference was found between the proportion of total CD3^+^CD4^+^ T helper cells in donor blood (63.5% [IQR 58.5, 65.5]) and the different PRP products (ACP®: 66.9% [IQR 60.5, 70.4]; Angel™: 65.0% [IQR 61.1, 68.0]; nSTRIDE® APS: 65.1% [IQR 62.2, 68.8]) (Fig. 1h). Accordingly, all PRP products also contained comparable levels of CD3^+^CD8^+^ cytotoxic T cells when compared to corresponding donor blood samples (donor blood: 32.1% [IQR 28.6, 33.1]; ACP®: 28.9% [IQR 25.3, 33.8; Angel™: 29.5% [IQR 28.7, 35.4]; nSTRIDE® APS: 30.0% [IQR 24.2, 33.1]) (Fig. 1i). The correlation analysis showed that relative amounts of CD3^+^CD4^+^ and CD3^+^CD8^+^ T cells within PRP products were donor-dependent and determined by the individual levels in the donor blood (Supplementary Fig. S4a-b). Correlation analyses of the CD4^+^ and CD8^+^ T cell subpopulations further showed that levels of naive, central memory, effector memory, and TEMRA T cells were also significantly correlated with the corresponding donor blood profile (Supplementary Figs. S5 and S6).

### PRP composition shows pro-inflammatory properties

To assess the cytokine profiles of the different PRP products in comparison to donor blood samples, levels of ten cytokines were analyzed as surrogate markers for the inflammatory composition of PRP products. In several samples of the PRP products and donor blood, the concentrations of IL-1β (donor blood: *n* = 4; ACP®: *n* = 6; Angel™: *n* = 8; nSTRIDE® APS: *n* = 6), IL-12p70 (donor blood: *n* = 1; ACP®: *n* = 1; nSTRIDE® APS: *n* = 2), and IL-13 (nSTRIDE® APS: *n* = 2) were below the limit of quantitation. Concentrations of IFN-γ, TFN-α, IL-2, IL-6, IL-8, IL4, and IL-10 were above the limit of detection in all samples (Table 1).

**Table 1:** Cytokine profile of donor blood and corresponding PRP samples

Cytokines	Donor blood	ACP®	Angel™	nSTRIDE® APS
IFN-γ [pg/ml]	1.765 (IQR 0.922, 3.999)	4.472 (IQR 3.174, 5.495)	3.832 (IQR 2.667, 4.706)	5.095 (IQR 3.357, 7.099)
TFN-α [pg/ml]	0.57 (IQR 0.316, 1.137)	0.988 (IQR 0.655, 1.33)	0.909 (IQR 0.652, 1.199)	1.264 (IQR 0.98, 1.523)
IL-1β [pg/ml]	0.417 (IQR 0.02, 0.708)	0.02 (IQR 0.02, 0.196)	0.02 (IQR 0.02, 0.041)	0.033 (IQR 0.02, 0.619)
IL-2 [pg/ml]	0.617 (IQR 0.234, 0.767)	0.557 (IQR 0.355, 0.959)	0.558 (IQR 0.424, 0.718)	0.315 (IQR 0.149, 0.581)
IL-6 [pg/ml]	0.355 (IQR 0.189, 0.52)	0.58 (IQR 0.293, 0.812)	0.478 (IQR 0.322, 0.636)	0.369 (IQR 0.25, 0.601)
IL-8 [pg/ml]	11.845 (IQR 2.504, 18.021)	7.139 (IQR 2.566, 8.48)	4.254 (IQR 2.507, 5.274)	7.089 (IQR 2.566, 26.494)
IL-4 [pg/ml]	0.057 (IQR 0.032, 0.134)	0.069 (IQR 0.008, 0.12)	0.041 (IQR 0.016, 0.08)	0.08 (IQR 0.056, 0.133)
IL-10 [pg/ml]	0.185 (IQR 0.128, 0.437)	0.367 (IQR 0.23, 0.507)	0.361 (IQR 0.234, 0.413)	0.312 (IQR 0.193, 0.473)
IL-12p70 [pg/ml]	0.201 (IQR 0.072, 0.359)	0.248 (IQR 0.038, 0.39)	0.143 (IQR 0.071, 0.311)	0.118 (IQR 0.038, 0.179)
IL-13 [pg/ml]	1.648 (IQR 0.934, 3.091)	1.985 (IQR 1.321, 2.235)	1.903 (IQR 1.151, 2.626)	1.079 (IQR 0.396, 1.847)

*Abbreviations*: *IFN-γ* interferon γ, *TNF-α* tumor necrosis factor α, *IL-1β* interleukin 1β, *IL-2* interleukin 2, *IL-4* interleukin 4, *IL-6* interleukin 6, *IL-8* interleukin 8, *IL-10* interleukin 10, *IL-12p70* interleukin 12p70, *IL-13* interleukin 13

**Fig. 2:**
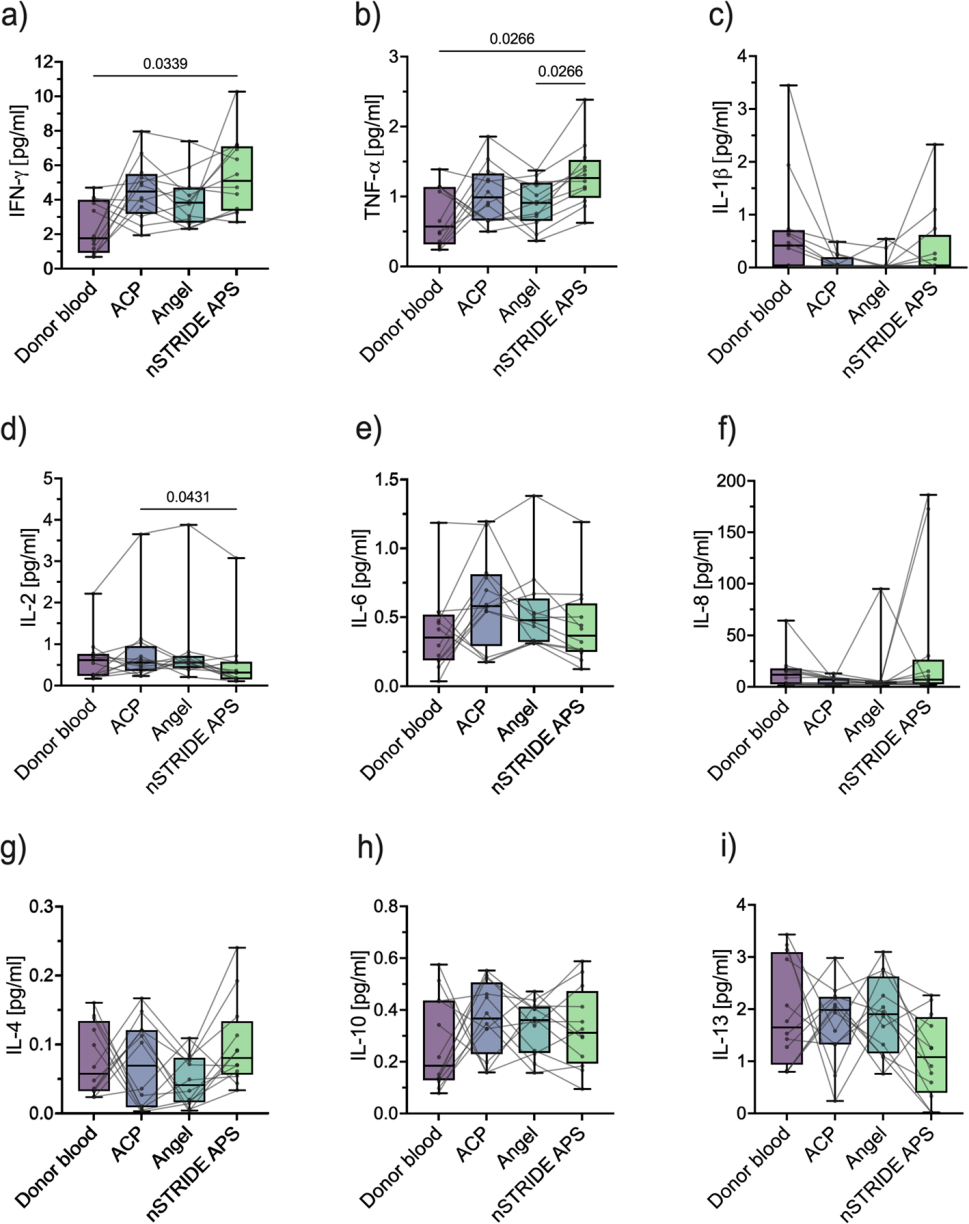
Cytokine profile of different PRP products and corresponding donor blood samples. **a** Concentrations of IFN-γ, **b** TNF-α, **c** IL-1β, **d** IL-2, **e** IL-6, **f** IL-8, **g** IL-4, **h** IL-10, and **i** IL-13 in donor blood compared to PRP samples. Abbreviations: IFN-γ, interferon γ; TNF-α, tumor necrosis factor α; IL-1β, interleukin 1β; IL-2, interleukin 2; IL-4, interleukin 4; IL-6, interleukin 6; IL-8, interleukin 8; IL-10, interleukin 10; IL-13, interleukin 13; PRP, platelet-rich plasma

The median IFN-γ concentration in the PRP products was approximately two to three times higher than in the donor blood samples, but reached statistical significance only between donor blood and nSTRIDE® APS samples (*p* = 0.034) (Fig. 2a). Significantly elevated TFN-α concentrations were also found in nSTRIDE® APS compared to donor blood (*p* = 0.027) and Angel™ samples (*p* = 0.027) (Fig. 2b). The median concentration of IL-2 was comparable between donor blood and the different PRP products, but significantly lower in nSTRIDE® APS compared to ACP® (*p* = 0.043) (Fig. 2d). The median concentrations of IL-1β, IL-6, IL-8, IL-4, IL-10, IL-12p70, and IL-13 were comparable between donor blood levels and the different PRP products without significant differences (Fig. 2c, e–i).

### IFN-γ and TNF-α influence population doubling dose-dependently

The role of inflammatory signals during healing is ambivalent [21, 22]. On the one hand, an initial pro-inflammatory response is essential to induce the regeneration of acute injuries; on the other hand, an extended inflammatory phase delays tissue repair [23]. Since some PRP products showed significantly increased levels of IFN-γ and TNF-α compared to donor blood, we investigated the influence of these cytokines on the proliferation and phenotype of primary human chondrocytes.

**Fig. 3:**
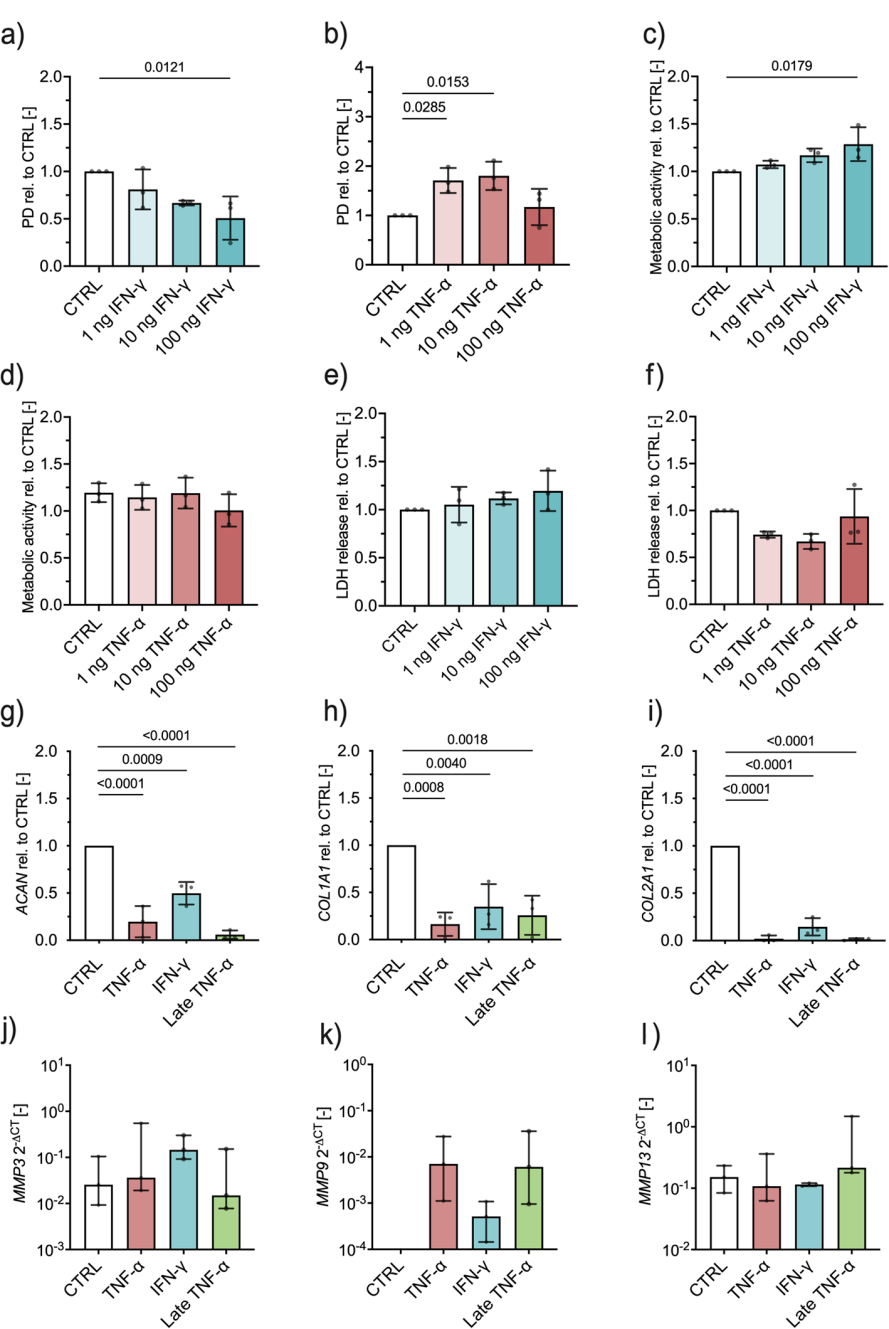
2D and 3D cell cultures of primary human chondrocytes exposed to a pro-inflammatory environment. **a** Population doublings dependent on IFN-γ and **b** TNF-α at indicated concentrations, **c** metabolic activity dependent on IFN-γ and **d** TNF-α at indicated concentrations, **e** lactate dehydrogenase (LDH) release dependent on IFN-γ and **f** TNF-α at indicated concentrations, fold change in mRNA expression relative to control of **g***ACAN*, **h***COL1A1*, and **i***COL2A1* and delta threshold cycles of **j***MMP3*, **k***MMP9*, and **l***MMP13* dependent on IFN-γ and TNF-α treatment, respectively. Abbreviations: PD, population doublings; CTRL, control; rel, relative; IFN-γ, interferon γ; TNF-α, tumor necrosis factor α; LDH, lactate dehydrogenase; *ACAN*, aggrecan; *COL1A1*, collagen type 1A1; *COL2A1*, collagen type 2A1; *MMP3*, matrix metalloproteinase 3; *MMP9*, matrix metalloproteinase 9; *MMP13*, matrix metalloproteinase 13; ∆CT, delta cycle threshold

Proliferation of chondrocytes was negatively influenced by higher concentrations of IFN-γ. While there was no significant difference in the number of PD between controls and 1 ng or 10 ng IFN-γ, the proliferation was significantly reduced in cultures treated with 100 ng IFN-γ compared to controls (*p* = 0.0121) (Fig. 3a). For TNF-α, we observed an opposite effect, as 1 ng (*p* = 0.0285) and 10 ng (*p* = 0.0153) TNF-α significantly enhanced cell proliferation compared to controls (Fig. 3b), the highest concentration of 100 ng reduced cell proliferation. The altered proliferation rates were not accompanied by a shift in metabolic activity. With the exception of 100 ng IFN-γ (*p* = 0.0179), even high cytokine concentrations had no influence on the metabolic activity compared to controls, thus ruling out apoptotic effects on chondrocytes (Fig. 3c, d). This was also confirmed by the quantification of LDH release as an indicator of apoptosis which showed no difference between cytokine-treated cultures and controls (Fig. 3e, f).

### Proteoglycan assay shows the development of cartilage in 3D chondrocyte cultures and is impaired when exposed to IFN-γ and TNF-α

To test the effect of IFN-γ and TNF-α on chondrocyte differentiation and their phenotype, we employed a previously described 3D disc assay [21]. Control cultures showed a significant deposition of proteoglycans in the extracellular matrix (ECM) as an indicator of successful cartilage formation. This effect was significantly impaired when discs were treated with TNF-α (*p* <0.0001) or IFN-γ (*p* = 0.0428) during chondrogenic differentiation (Supplementary Fig. S7). To evaluate the effects of TNF-α on the chondrocyte phenotype, late TNF-α cultures were first differentiated for 14 days without inflammatory cytokine supplementation and then subsequently stimulated with TNF-α for another 14 days. Late TNF-α cultures also exhibited significantly lower proteoglycan content compared to control cultures. This experiment showed that TNF-α not only inhibited the differentiation of chondrocytes, but also negatively affected the phenotype of already differentiated cells. To further test this finding, we analyzed mRNA expression of key factors involved in cartilage formation, maintenance, and destruction upon stimulation with IFN-γ and TNF-α. In line with the proteoglycan data, we found that mRNA expression of *ACAN* (TNF-α: *p* <0.0001; IFN-γ: *p* = 0.0009; late TNF-α: *p* <0.0001), *COL1A1* (TNF-α: *p* = 0.0008; IFN-γ: *p* = 0.0040; late TNF-α: *p* = 0.0018), and *COL2A1* (TNF-α: *p* <0.0001; IFN-γ: *p* <0.0001; late TNF-α: *p* <0.0001) were significantly decreased in cultures exposed to TNF-α and IFN-γ compared to controls after 28 days of culture (Fig. 3g, h, and i). Interestingly, the expression of *MMP3*, *MMP9*, and *MMP13* was very low or even below the detection limit in controls, but detectable in some IFN-γ-stimulated cultures and significantly increased in all cultures treated with TNF-α, indicating a robust catabolic response towards pro-inflammatory signals (Fig. 3j, k, l). Collectively, these results prove that TNF-α and IFN-γ negatively affect the phenotype and function of chondrocytes.

## Discussion

Despite the constantly rising number of patients suffering from OA [24], there is, thus far, no causal treatment for OA and symptomatic treatment cannot halt disease progression [25]. The local low-grade inflammation in OA has been linked to cartilage degeneration and subsequent joint destruction [26]. Complementary to surgical treatment strategies [27], various potentially disease-modifying drugs that may selectively disrupt inflammatory pathways present in OA are currently being investigated [28]. Of these, PRP is one of the most popular products already in clinical use. PRP was observed to be superior to other therapeutics, including hyaluronic acid, corticosteroids, and placebo, with respect to clinical outcomes and disease progression in OA [29, 30]. However, response to therapy is highly heterogenous and PRP fails in a relevant proportion of affected patients. To date, reasons for absent responses to treatment remain elusive.

To the best of our knowledge, this is the first study assessing cellular and cytokine compositions of various commercially available PRP systems used in daily clinical practice and comparing these to corresponding donor blood samples.

All PRP systems resulted in a significant proportional enhancement of leukocytes. This observation is consistent with previous findings [31–38]. Commercially available PRP systems result in products with varying leukocyte concentrations that can be categorized into two groups: leukocyte-rich PRP (LR-PRP) and leukocyte-poor PRP (LP-PRP). While LR-PRP systems tend to aggregate leukocytes, LP-PRP systems reduce leukocyte concentrations compared to corresponding blood concentrations [31–38]. Slow spin speeds of around 1500 rpm are associated with an up to threefold concentration of platelets, an almost complete elimination of red blood cells (RBC), and a reduction of leukocyte concentrations (LP-PRP), whereas higher spin speeds of around 3200 rpm are associated with an up to ninefold concentration of platelets, some loss of RBCs, and an increase in leukocyte concentrations (LR-PRP) [31]. Previous studies ranked the ACP® and the Angel™ systems among the LP-PRP systems [2, 37, 39].

The concentration of leukocytes was accompanied by an overall reduction in granulocytes and a proportional increase of lymphocytes and monocytes. Donor blood samples contained 1.1% of leukocytes, of which over 60% were granulocytes, which was reduced to at least 40% in final PRP products. The lymphocyte and monocyte proportion was increased from around 40% in donor blood to around 60% and above in the PRP samples. As previously observed [31], the nSTRIDE® APS system, categorized as an LR-PRP system, showed higher proportions of granulocytes when compared to the LP-PRP systems, ACP® and Angel™. Wakayama et al. compared the nSTRIDE® APS and the MyCells® (LP-)PRP system. Both systems concentrated lymphocytes, but only the nSTRIDE® system concentrated neutrophils in both healthy volunteers and OA patients [34]. Fitzpatrick et al. compared the GPS III, SmartPrep® II, and the ACP®. They detected that neutrophils and lymphocytes were the most concentrated fractions within the leukocyte populations [37]. These results were supported by a canine feasibility study that solely analyzed the nSTRIDE® APS system and observed a global concentration of leukocytes, neutrophils, lymphocytes, and monocytes [35]. The aforementioned findings are not completely in accordance with our data, but only Wakayama et al. reported data on human subjects [34]. As the proportion of neutrophils and monocytes directly influences the composition of pro- and anti-inflammatory cytokines in the final product [31], this may impact clinical outcomes in treated patients.

We observed considerably varying cytokine compositions between products. The concentrations of the pro-inflammatory cytokines IFN-γ and TNF-α were significantly increased in the nSTRIDE® APS when compared to donor blood samples. This was not the case for the other two systems. As PRP is mainly used for its anti-inflammatory properties, this finding appears counterintuitive. Previous studies have observed a concentration of pro-inflammatory cytokines, such as IL-1β, IL-6, and TNF-α, especially in LR-PRP products [33, 34, 38–40]. Increased levels of IL-1β and TNF-α directly contribute to cartilage degradation and enhanced IL-6 production, which then enhances inflammatory responses that cause bone resorption in OA [41]. Accordingly, most authors do not recommend the use of LR-PRP for OA therapy, as they expect these products to increase or, at least, maintain the low-grade inflammation present in affected joints [38, 42, 43]. In laboratory studies, however, LR-PRP prevented chondrogeneous matrix degradation, increased chondrocyte cellularity, and inhibited the production of MMP13 [44–46]. Interestingly, both Mariani et al. and Cole et al. observed no local changes in cytokine concentrations in synovial fluid after PRP injections [39, 47]. These observations are confirmed by clinical studies, which showed the overall effectiveness of products with various cytokine compositions, including LR-PRP, for OA treatment [48]. While the relevance of cytokine compositions in PRP products on clinical outcomes in OA may be questioned, it is important to note that regardless of individual donor blood cytokine compositions, some PRP products will yield a high pro-inflammatory cytokine composition, which may affect immune cells already present in the affected joint.

When comparing donor blood and corresponding PRP samples, we observed a maintenance of each participant’s adaptive immune profile. This is highly relevant, since high systemic levels of distinct T cell subsets have been associated with impaired tissue healing [23, 49]. These TEMRA T cells accumulate in large numbers also at the injury site and are the major local producers of pro-inflammatory cytokines [23], which are, again, linked to the age-related phenotype [50] and the development of OA [51]. Furthermore, a local downregulation of these cells led to a decreased concentration of pro-inflammatory cytokines and an improved bone regeneration in a preclinical fracture model [52]. Our observations could help to understand the heterogeneous clinical effectiveness of PRP in OA therapy, as we observed that the individual adaptive immune profile is directly transferred into the PRP products. Further clinical studies are needed to examine this potential influence of patients’ individual adaptive immune system and its receptivity to regenerative therapies, such as PRP.

To assess the impact of the observed cytokine compositions of different PRP products on cartilage, we chose the most dominant pro-inflammatory cytokines IFN-γ and TNF-α which were co-cultured with primary human chondrocytes. While the viability and general metabolic activity of chondrocytes were not affected, we observed a dose-dependent change in proliferation rates. While TNF-α treatment was beneficial regarding cell proliferation, high IFN-γ concentrations resulted in decreased PD. In general, increased proliferation is commonly associated with decreased differentiation capacity and can, thus, be the cause for dysfunctional tissue regeneration and function [23]. Adding inflammation to an existing pro-inflammatory condition, as seen in OA, might, therefore, lead to further local cartilage degeneration.

When assessing mRNA expression levels of relevant cartilage markers as a response to pro-inflammatory cytokine exposure, we observed a striking effect on markers specific for mature and functional cartilage. For example, *ACAN* expression was significantly reduced under all pro-inflammatory stimuli. Aggrecan is highly relevant for the structural integrity and function of mature cartilage and one of the earliest markers for the onset of OA [53]. The same was observed for *COL1A1* and *COL2A1*, where IFN-γ and TNF-α treatment significantly reduced the expression of these ECM components, which are essential for chondrogenesis and maintenance of mature cartilage. Even after chondrogenic differentiation, TNF-α was able to degrade and impair functional cartilage when added to the culture after 14 days. This effect indicates that prolonged inflammation can affect functional cartilage and hinder regeneration, which has already been described for fracture and muscle repair [54].

To confirm this negative impact, the mRNA expression of *MMP3*, *MMP9*, and *MMP13* was determined. We observed no expression of these genes in untreated controls but a strong increase upon stimuli with IFN-γ and TNF-α. This increase of catabolic enzymes further strengthens the concept that chondrocyte malfunctioning and cartilage degeneration can be initiated by prolonged exposure to inflammation. *MMP3* degrades several ECM components, activates other proteolytic enzymes, and is elevated in different pathogeneses of cartilage degeneration [55]. *MMP9* and *MMP13* expressions are also elevated in cartilage pathologies as well as in synovial inflammation and have been discussed as biomarkers for OA [56–58]. This is also supported by the impaired proteoglycan deposition and ECM formation following IFN-γ and TNF-α stimulation in 3D cartilage discs. Collectively, these results provide evidence that IFN-γ and TNF-α negatively affect the phenotype and function of chondrocytes. This supports our presumption that clinical PRP effectiveness is dependent on individual product composition.

This study has some limitations. First, we provided data from a rather small cohort of healthy participants. While PRP is predominantly an autologous treatment concept, allogenic (homologous) PRP applications have shown promising results in elderly patients with knee OA [59] and might be applicable to patients who are not eligible for autologous treatments (poor general health, multidrug therapy, hematologic disorders) [59, 60]. Furthermore, immunological reactions were not observed in preclinical assessments, following autologous PRP infiltration of bone and muscle in rabbits [61]. There is no clear evidence for a negative correlation between age and “potency” of PRP, yet it was shown that the composition of PRP of young donors differs from that of aged donors [62–64].

Second, correlations of our results with clinical outcome data are not available. In the future, prospective observational or randomized interventional studies are needed in order to analyze our findings for clinical relevance. On the other hand, this is the first study to merge the cellular and cytokine compositions of different PRP products and match these to individual donor immune profiles. This is highly relevant, as authors were previously able to highlight the influence of the individual adaptive immune capacities on tissue regeneration [23, 52]. The systems used for PRP production in our study are commercially available and broadly used in clinical practice, allowing for a high comparability with other researchers’ results.

## Conclusion

All PRP systems examined significantly enhanced leukocytes, which was accompanied by an overall reduction in granulocytes and a proportional increase of lymphocytes and monocytes. Furthermore, each participant’s adaptive immune profile was maintained in the final PRP products and the cytokine compositions highly varied between products. Pro-inflammatory cytokines negatively affected the phenotype and function of chondrocytes. These observations may help to answer why patients benefit differently from PRP treatment in OA.

## Supplementary Information

**Additional file 1: Supplementary Table S1.** Primer list. Abbreviations: HGPRT: Hypoxanthine-guanine phosphoribosyltransferase, *ACAN*: Aggrecan, *COL1A1*: Collagen type 1A1, *COL1A2*: Collagen type 2A1, *MMP3*: Matrix metalloproteinase 3, *MMP9*: Matrix metalloproteinase 9, *MMP13*: Matrix metalloproteinase 13.

**Additional file 2: Supplementary Figure S1.** Gating strategy derived from Kaluza Analysis. a) Gating strategy for basic characterization, and b) for T cell subset characterization.

**Additional file 3: Supplementary Figure S2. **Portion of monocyte subsets in the PRP and donor blood samples. a) displays classical (CD45^+^CD14^high^CD16^−^), b) non-classical (CD45^+^CD14^dim^CD16^+^), and c) intermediate subgroup of monocytes (CD45^+^CD14^high^CD16^+^).

**Additional file 4: Supplementary Figure S3.** Correlation analysis of B, T, and NK cells. a) CD19^+^ B cells (ACP®: r = 0.937, *p* < 0.0001; Angel™: r = 0.961, *p* < 0.0001; nSTRIDE® APS: r = 0.847, *p* = 0.0005), b) CD3^+^ T cells (ACP®: r = 0.73, *p* = 0.007; Angel™: r = 0.558, *p* = 0.06; nSTRIDE® APS: r = 0.108, *p* = 0.739), and c) CD3^-^CD56^+^ NK cells (ACP®: r = 0.944, *p* < 0.0001; Angel™: r = 0.629, *p* = 0.028; nSTRIDE® APS: r = 0.368, *p* = 0.239). Abbreviations: NK cells: Natural killer cells.

**Additional file 5: Supplementary Figure S4.** Correlation analysis of a) CD4^+^ and b) CD8^+^ T cells in donor blood and corresponding PRP samples.

**Additional file 6: Supplementary Figure S5.** Correlation analysis of CD4^+^ T cell substes, including central memory (a), naive (b), effector memory (c), and TEMRA (d) T cells in donor blood and corresponding PRP samples.

**Additional file 7: Supplementary Figure S6.** Correlation analysis of CD8^+^ T cell substes, including central memory (a), naive (b), effector memory (c), and TEMRA (d) T cells in donor blood and corresponding PRP samples.

**Additional file 8: Supplementary Figure S7.** Proteoglycan assay of cartilage in 3D chondrocyte cultures exposed to IFN-γ and TNF-α. Proteoglycan content normalized to total protein content showed a reduction following exposure to TNF-α (*p* <0.0001) and IFN-γ (*p* = 0.0428) relative to control.

## Data Availability

The data sets used and/or analyzed in the present study are available from the corresponding author upon request.

## Abbreviations

*ACAN*: Aggrecan
ACD: Acid-citrate-dextrose
BSA: Bovine serum albumin
CD: Cluster of differentiation
CDM: Chondrogenic differentiation medium
*COL1A1*: Collagen type 1A1
*COL2A1*: Collagen type 2A1
DMMB: Dimethylmethylene blue
EDTA: Ethylenediaminetetraacetic acid
ELISA: Enzyme-linked immunosorbent assay
FBS: Fetal bovine serum
FDA: Food and Drug Administration
LDH: Lactate dehydrogenase
IFN-γ: Interferon gamma
LG-DMEM: Low-glucose Dulbecco’s modified Eagle medium
IL-1β: Interleukin 1β
IL-2: Interleukin 2
IL-4: Interleukin 4
IL-6: Interleukin 6
IL-8: Interleukin 8
IL-10: Interleukin 10
IL-12p70: Interleukin 12p70
IL-13: Interleukin 13
IQR: Interquartile range
ITS: Insulin-transferrin-sodium selenite media supplement
MSD: Meso Scale Discovery
*MMP3*: Matrix metalloproteinase 3
*MMP9*: Matrix metalloproteinase 9
*MMP13*: Matrix metalloproteinase 13
NGr: Neutrophil granulocytes
OA: Osteoarthritis
PBS: Phosphate-buffered saline
NK cells: Natural killer cells
PD: Population doublings
PRP: Platelet-rich plasma
*p*-value: Probability value
qPCR: Real-time quantitative polymerase chain reaction
RPM: Revolutions per minute
RNA: Ribonucleic acid
TEMRA: Terminally differentiated effector memory
TGF- β1: Transforming growth factor β1
TNF-α: Tumor necrosis factor alpha
2D: Two dimensional
3D: Three dimensional
