# Supplementary material for: Individual immune cell and cytokine profiles determine platelet-rich plasma composition
Source: Arthritis Res Ther. 2023 Jan 10;25:6. doi: 10.1186/s13075-022-02969-6 (PMC9830842; doi:10.1186/s13075-022-02969-6)
Supplement: Supplementary file 1 — Additional file 1: Supplementary Table S1. Primer list. Abbreviations: HGPRT: Hypoxanthine-guanine phosphoribosyltransferase, ACAN: Aggrecan, COL1A1: Collagen type 1A1, COL1A2: Collagen type 2A1, MMP3: Matrix metalloproteinase 3, MMP9: Matrix metalloproteinase 9, MMP13: Matrix metalloproteinase 13. [file 13075_2022_2969_MOESM1_ESM.docx]

| **Supplementary Table S1**. Primer list. |  |
| --- | --- |
| Gene | Sequence 5' - 3' |
| HGPRT | TATGGACAGGACTGAACGTC |
|  | TGATGTAATCCAGCAGGTCA |
| *ACAN* | GGGTTTTCGTGACTCTGAGG |
|  | ATGGGGTCGATGAAATAGCA |
| *COL2A1* | AGCCGGAGATAGAGGACCAC |
|  | GGCCAAGTCCAACTCCTTTT |
| *COL1A1* | CTGGAAAAGATGGTCCCAAA |
|  | CAGGGAATCCTCTCTCACCA |
| *MMP3* | TTGAGCTGGACTCATTGTCG |
|  | TCTCGGAGCCTCTCAGTCAT |
| *MMP9* | TGACAGCGACAAGAAGTGGG |
|  | TTCAGGGCGAGGACCATAGA |
| *MMP13* | TTGAGCTGGACTCATTGTCG |
|  | TCTCGGAGCCTCTCAGTCAT |
| Abbreviations: HGPRT: Hypoxanthine-guanine phosphoribosyltransferase, *ACAN*: Aggrecan, *COL1A1*: Collagen type 1A1, *COL1A2*: Collagen type 2A1, *MMP3*: Matrix metalloproteinase 3, *MMP9*: Matrix metalloproteinase 9, *MMP13*: Matrix metalloproteinase 13. | |
