# Supplementary figures and images for: Individual immune cell and cytokine profiles determine platelet-rich plasma composition
Source: Arthritis Res Ther. 2023 Jan 10;25:6. doi: 10.1186/s13075-022-02969-6 (PMC9830842; doi:10.1186/s13075-022-02969-6)

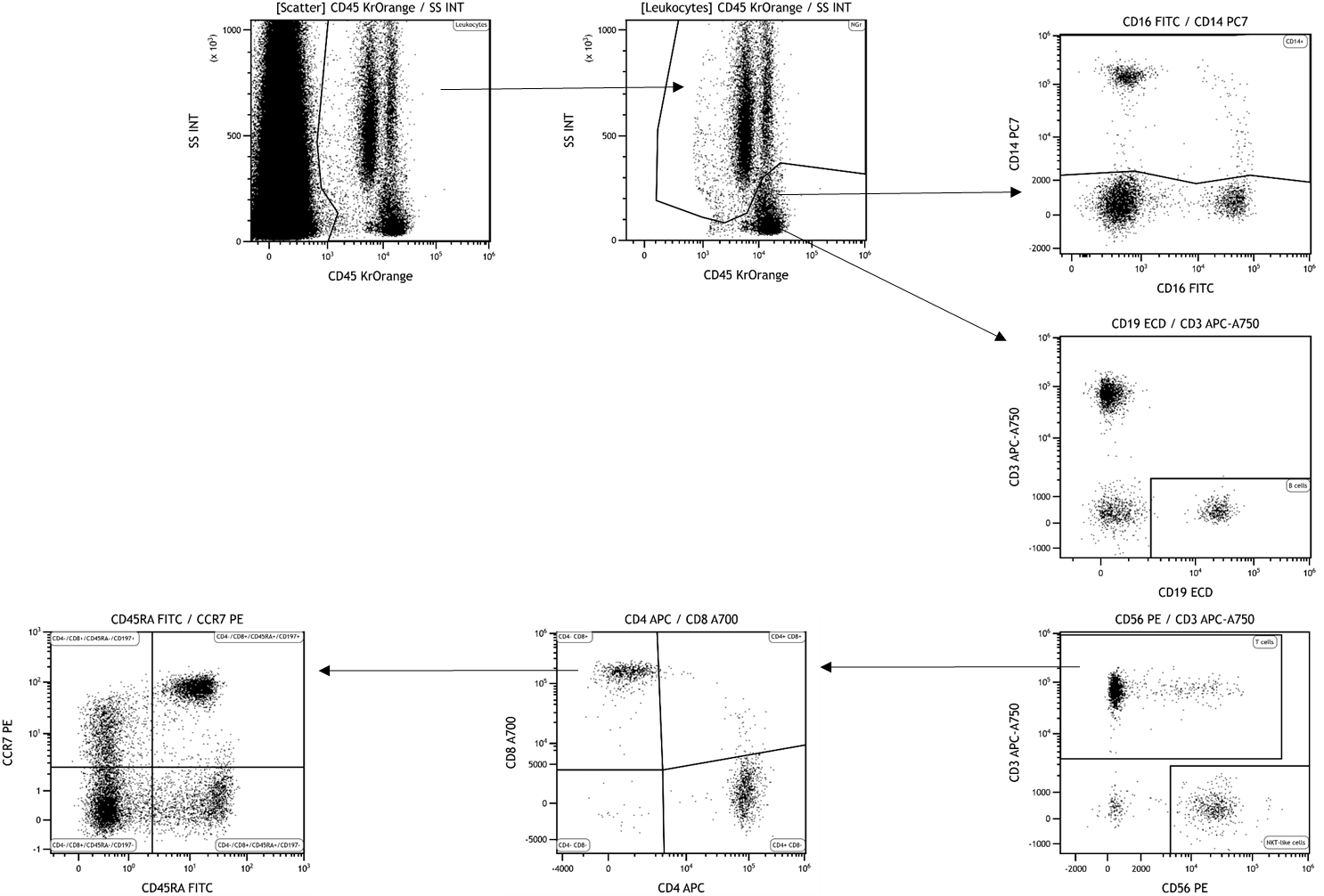

Supplement: Supplementary file 2 — Additional file 2: Supplementary Figure S1. Gating strategy derived from Kaluza Analysis. a) Gating strategy for basic characterization, and b) for T cell subset characterization. [file 13075_2022_2969_MOESM2_ESM.tiff]

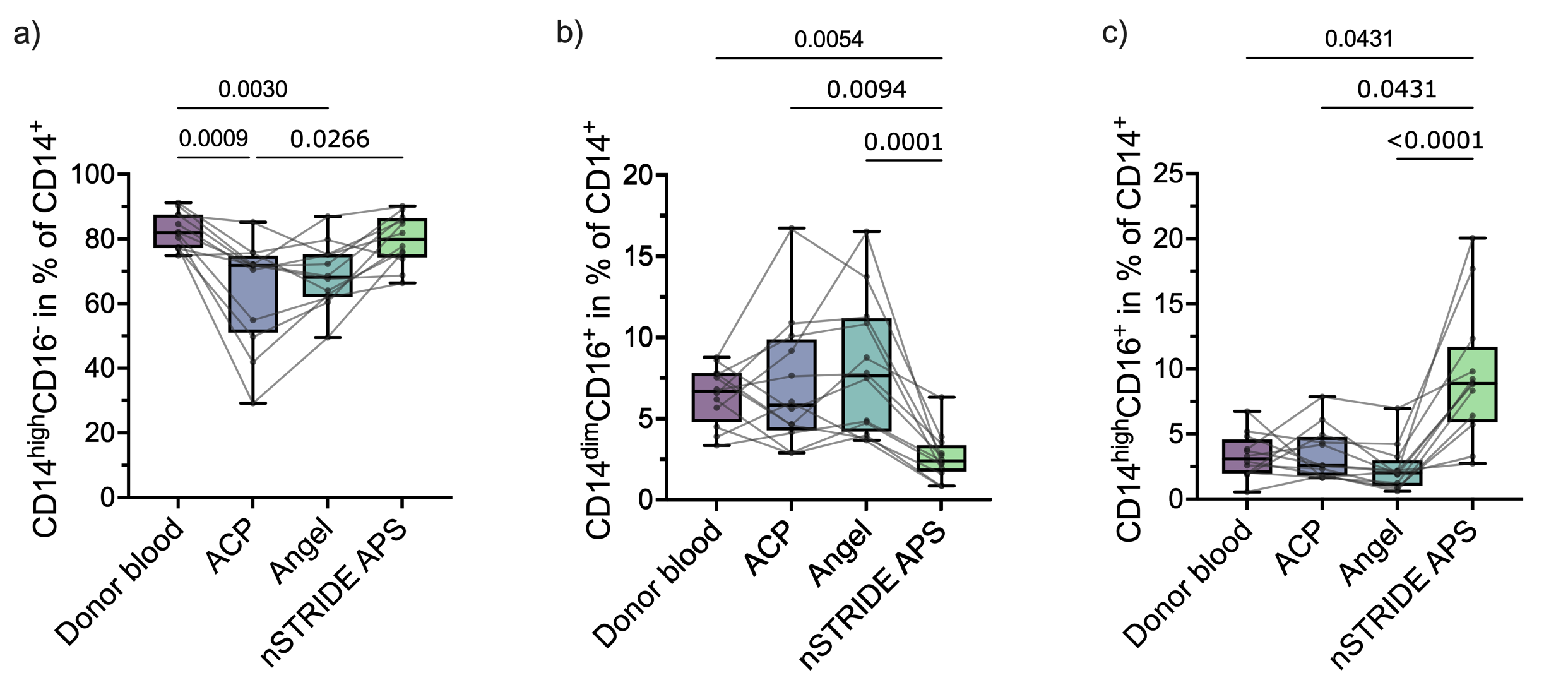

Supplement: Supplementary file 3 — Additional file 3: Supplementary Figure S2. Portion of monocyte subsets in the PRP and donor blood samples. a) displays classical (CD45+CD14highCD16−), b) non-classical (CD45+CD14dimCD16+), and c) intermediate subgroup of monocytes (CD45+CD14highCD16+). [file 13075_2022_2969_MOESM3_ESM.tiff]

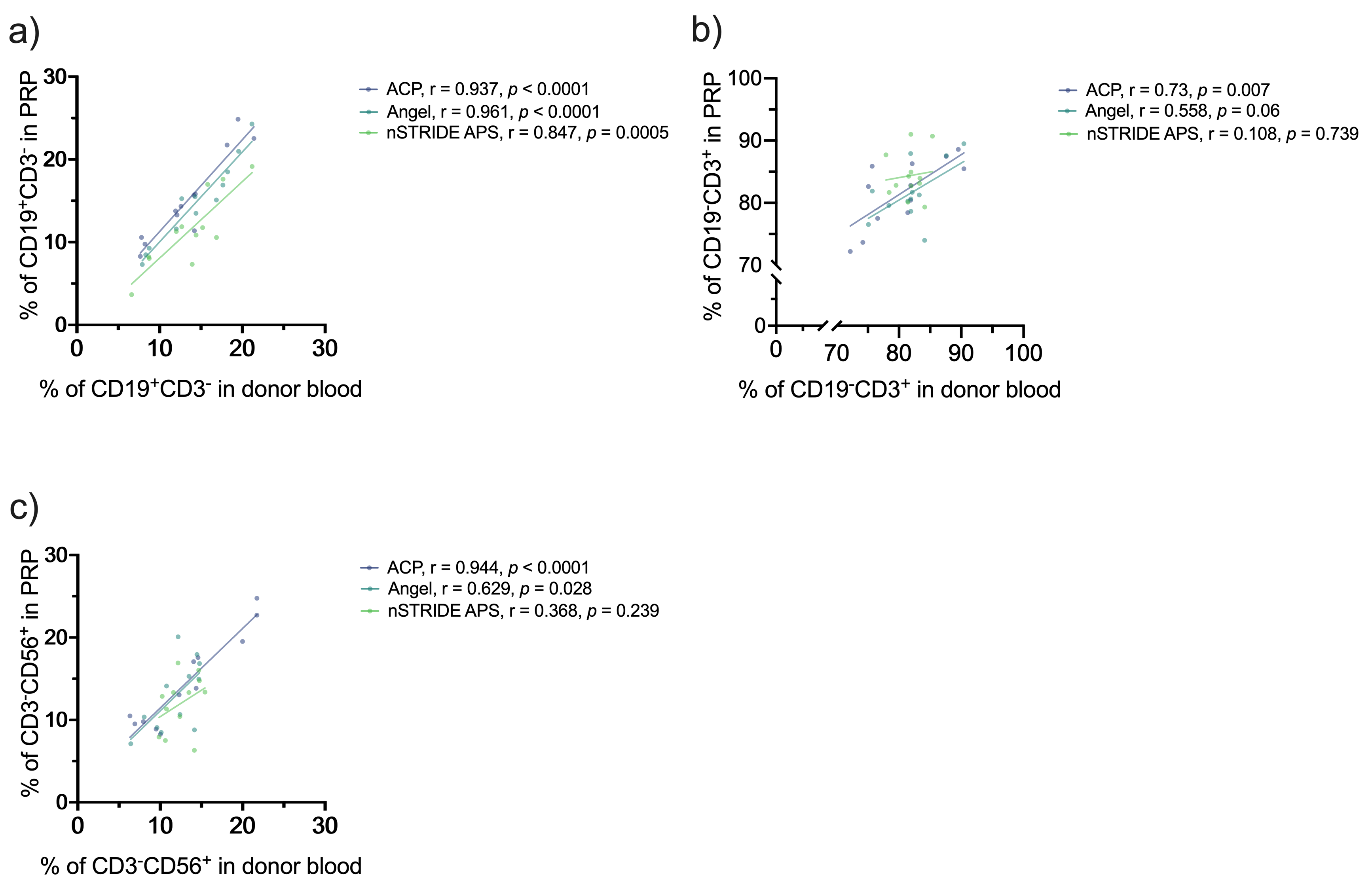

Supplement: Supplementary file 4 — Additional file 4: Supplementary Figure S3. Correlation analysis of B, T, and NK cells. a) CD19+ B cells (ACP®: r = 0.937, p < 0.0001; Angel™: r = 0.961, p < 0.0001; nSTRIDE® APS: r = 0.847, p = 0.0005), b) CD3+ T cells (ACP®: r = 0.73, p = 0.007; Angel™: r = 0.558, p = 0.06; nSTRIDE® APS: r = 0.108, p = 0.739), and c) CD3-CD56+ NK cells (ACP®: r = 0.944, p < 0.0001; Angel™: r = 0.629, p = 0.028; nSTRIDE® APS: r = 0.368, p = 0.239). Abbreviations: NK cells: Natural killer cells. [file 13075_2022_2969_MOESM4_ESM.tiff]

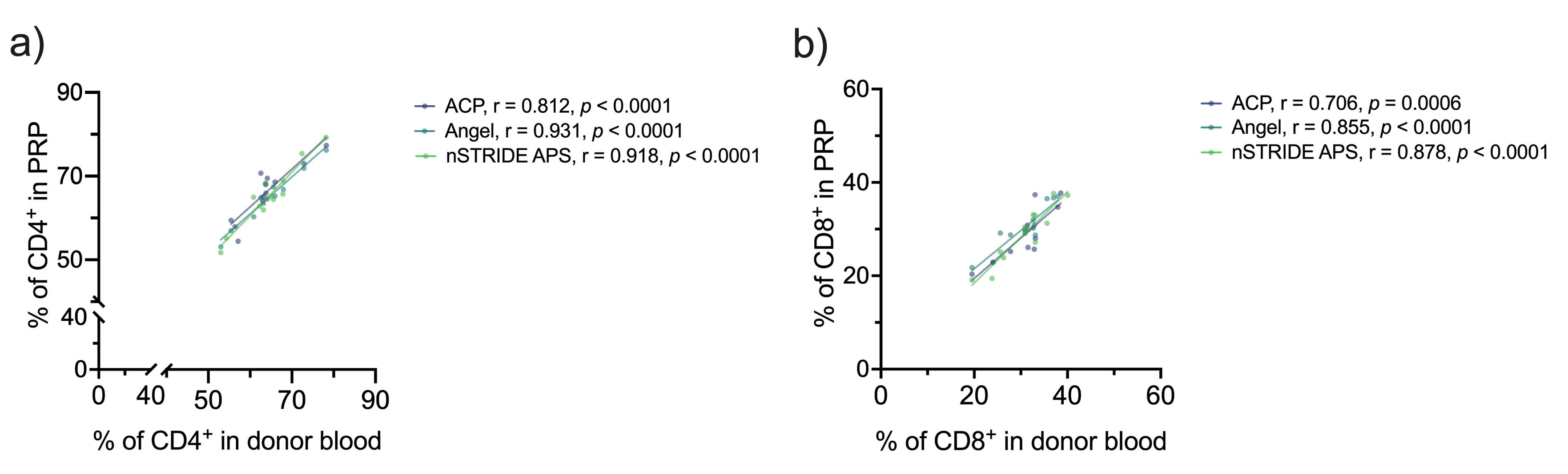

Supplement: Supplementary file 5 — Additional file 5: Supplementary Figure S4. Correlation analysis of a) CD4+ and b) CD8+ T cells in donor blood and corresponding PRP samples. [file 13075_2022_2969_MOESM5_ESM.tiff]

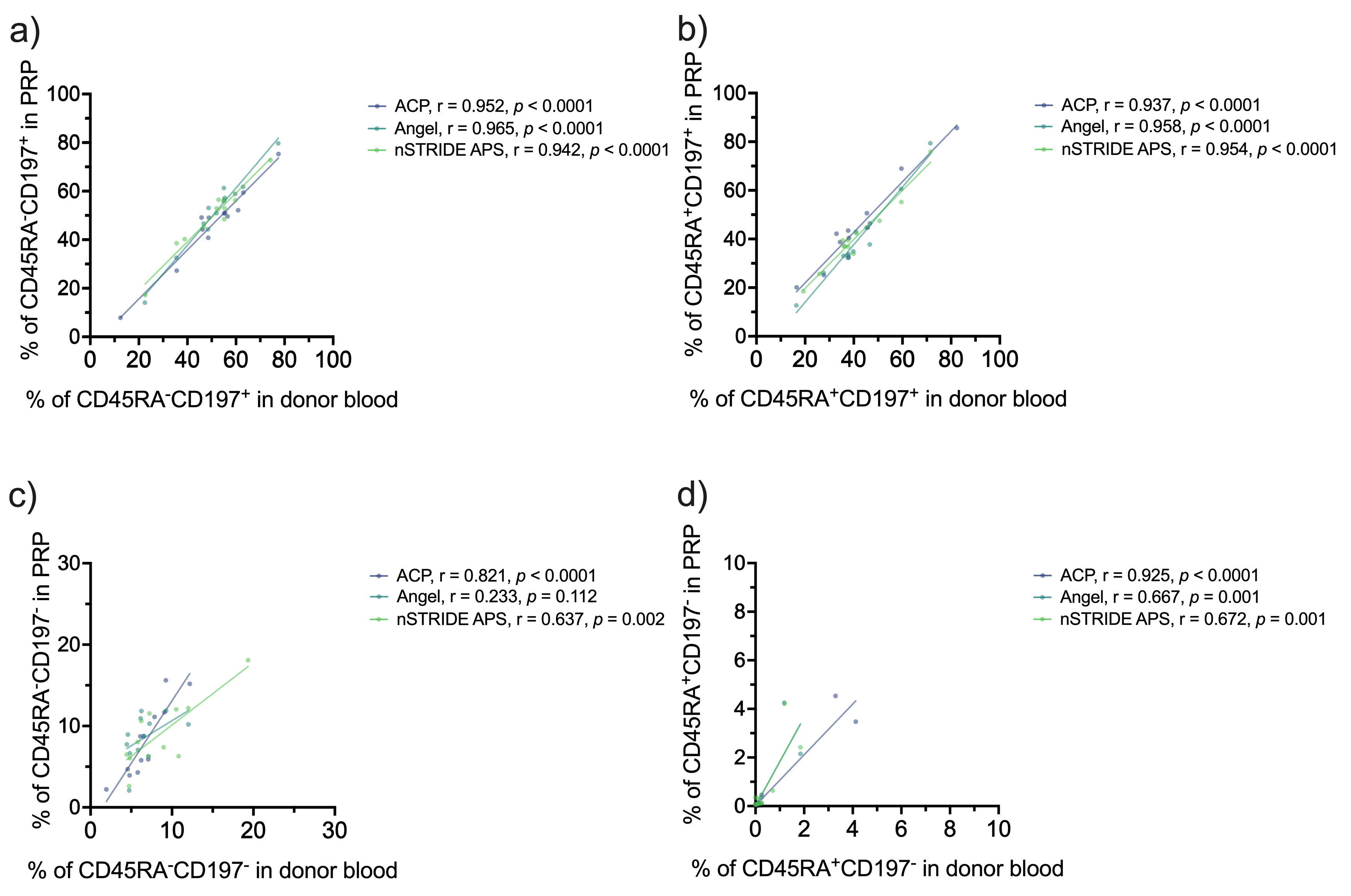

Supplement: Supplementary file 6 — Additional file 6: Supplementary Figure S5. Correlation analysis of CD4+ T cell substes, including central memory (a), naive (b), effector memory (c), and TEMRA (d) T cells in donor blood and corresponding PRP samples. [file 13075_2022_2969_MOESM6_ESM.tiff]

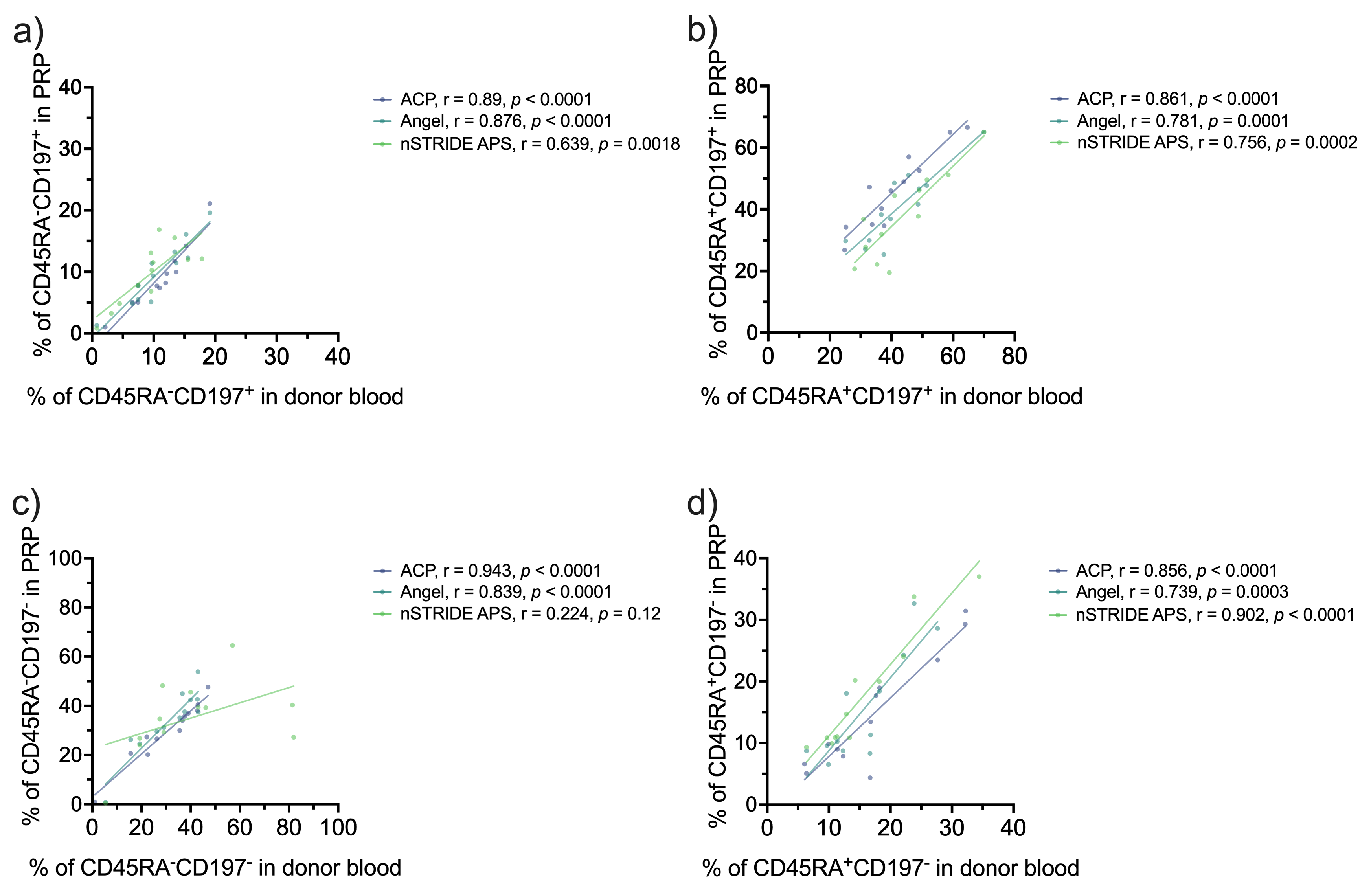

Supplement: Supplementary file 7 — Additional file 7: Supplementary Figure S6. Correlation analysis of CD8+ T cell substes, including central memory (a), naive (b), effector memory (c), and TEMRA (d) T cells in donor blood and corresponding PRP samples. [file 13075_2022_2969_MOESM7_ESM.tiff]

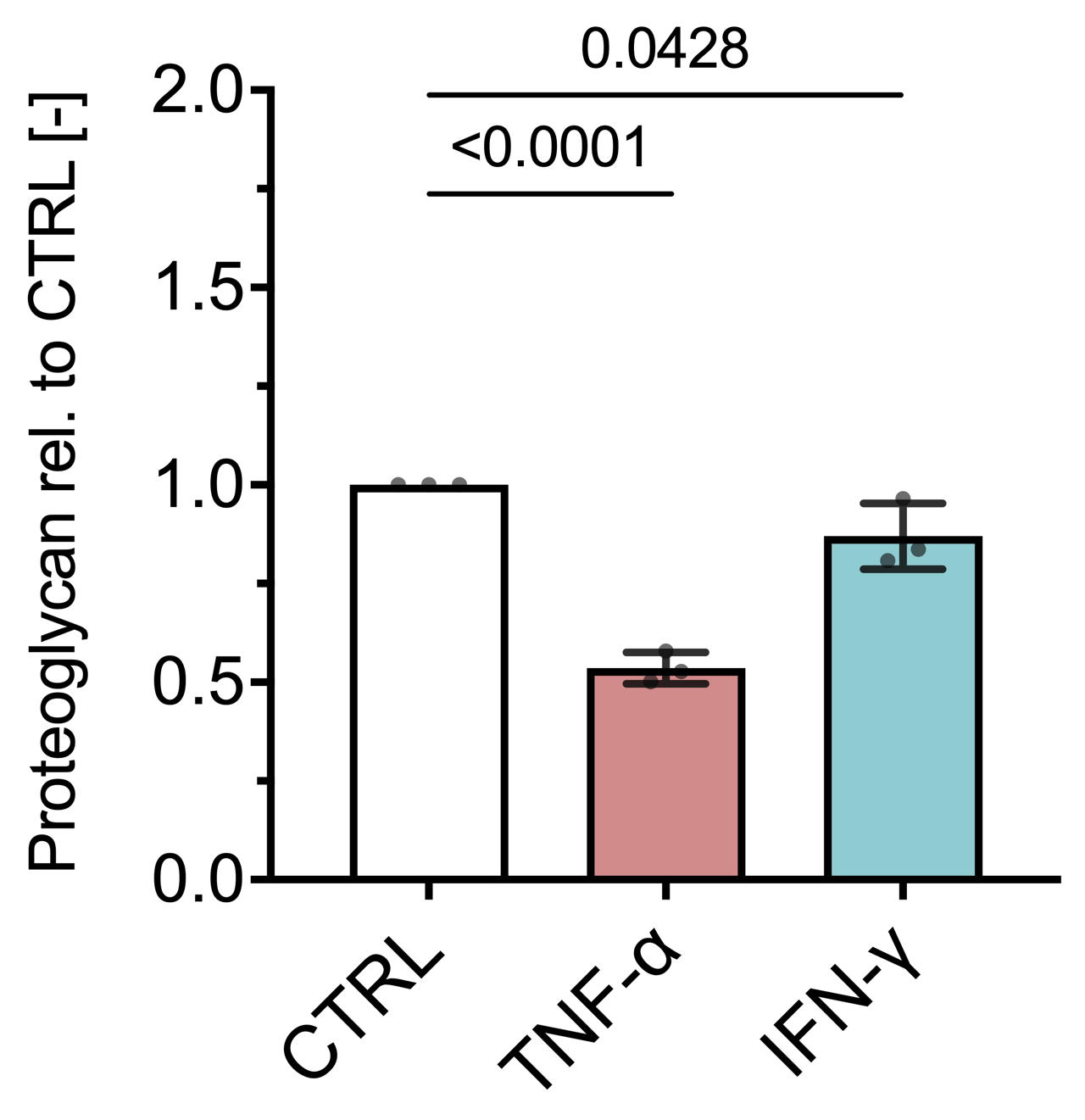

Supplement: Supplementary file 8 — Additional file 8: Supplementary Figure S7. Proteoglycan assay of cartilage in 3D chondrocyte cultures exposed to IFN-γ and TNF-α. Proteoglycan content normalized to total protein content showed a reduction following exposure to TNF-α (p <0.0001) and IFN-γ (p = 0.0428) relative to control. [file 13075_2022_2969_MOESM8_ESM.tiff]
